# Root Canal Disinfection Articles with the Highest Relative Citation Ratios. A Bibliometric Analysis from 1990 to 2019

**DOI:** 10.3390/antibiotics10111412

**Published:** 2021-11-18

**Authors:** Pilar Valderrama, Pilar Baca, Carmen Solana, Carmen María Ferrer-Luque

**Affiliations:** 1 Department of Information and Communication, University of Granada, 18071 Granada, Spain; piluvb95@ugr.es; 2 Department of Stomatology, University of Granada, 18071 Granada, Spain; cmsolana4@gmail.com (C.S.); cferrer@ugr.es (C.M.F.-L.)

**Keywords:** bibliometrics, citations, endodontics, National Institutes of Health, relative citation ratio, root canal disinfection, citation impact, article-level, field-normalization, time-normalization

## Abstract

The relative citation rate (RCR) is a normalized article-level metric useful to assess the impact of research articles. The objective of this bibliometric study is to identify and analyze, in root canal disinfection, the 100 articles having the highest RCRs in the period 1990–2019, then compare them with the top 100 articles most cited. A cross-sectional study was performed, and the search strategy ((Disinfection AND root canal) AND ((“1990/01/01”[Date-Publication]: “2019/12/31”[Date-Publication]))) relied on PubMed (*n* = 4294 documents), and article data were downloaded from the iCite database. The 100 articles with the highest RCRs and the top 100 cited were selected and evaluated in bibliometric terms. Among the 100 articles with the highest RCRs, there were no differences in the three decades for RCRs values, but there were in citations, being 2000–2009 the most cited. The USA was the predominant country (*n* = 30), followed by Brazil (*n* = 14). The most frequent study designs were reviews (*n* = 27) and in vitro (*n* = 25) and ex vivo (*n* = 24) studies. All subfields were well represented, although they varied over time. In 2010–2019, regenerative procedures and irrigation/disinfection techniques were predominant. Considering the RCR’s top 100 articles, 76 were common with the 100 most cited articles. Using the RCR metric allowed us to identify influential articles in root canal disinfection, a research field with topics of significance that fluctuate over time. Compared to citations, RCR reduces the time from publication to detection of its importance for the readership and could be a valid alternative to citation counts.

## 1. Introduction

Bacteria and their by-products are the etiologic reasons for pulpal and periapical diseases. However, the complete elimination of microorganisms from the root canal system looms as a challenging task to achieve [1]. Thus, the focus should be on reducing intracanal bacterial populations to levels that are compatible with periapical tissue healing [2], avoiding adverse effects as much as possible [3]. Apart from the control of bacteria, other factors—a correct diagnosis, adequate chemomechanical preparation, and hermetic tridimensional obturation—are necessary for successful endodontic treatment.

Because mechanical instrumentation alone is insufficient to yield effective disinfection [4], chemical irrigation with antibacterial and chelating agents is crucial for decreasing the bacterial population in standard irrigation regimes [5]. To enhance disinfection in secondary endodontic infections with more elaborate microbiota [6], or in cases involving a more complex anatomy of the root canal system [7], conventional treatment would not suffice to provide an adequate disinfection environment [1]. The use of intracanal medication with antimicrobial activity between sessions has been recommended to eliminate possibly persistent microorganisms and/or intracanal exudates, thus constituting an additional strategy in all the above cases, as well as in regenerative endodontics [8]. Such procedures commonly involve a mixture of antibiotics in a paste as intracanal medication [9]. In addition, irrigating/disinfection techniques, e.g., sonically activated irrigation, high-power lasers, and antimicrobial photodynamic therapy, among others, can contribute to achieving enhanced disinfection [10].

Bibliometrics stands as a quantitative analytical and statistical field with a focus on research papers published in scientific journals and their citations. Bibliometric analysis serves to evaluate various aspects of scientific articles, perhaps to reveal the historical development of research fields, or else to evaluate the scientific productivity of researchers, organizations, countries, institutions, and journals [11]. Citation analysis is one of the most frequently used methods for quantifying and comparing the impact of scholarly articles, distinguishing most-cited articles, visualizing trending topics, and recording “citation classics” (over 100 cites) across subject categories [12,13].

Bibliometric analysis may be used by researchers to examine and assess publications on medical research, including fields such as infectious diseases [14,15,16,17] or dentistry [18,19,20,21]. In recent years, endodontics has also been the object of Scientometric and/or bibliometric studies, analyzing scientific output globally [22], or in a specific field, such as regenerative endodontics [23], micro-CT [24], or vital pulp therapy [25]. Bibliometrics has also been used to identify and characterize the most-cited articles published in journals of endodontics [26,27] or circumscribed only to the leaders in the area, i.e., Journal of Endodontics [28], International Endodontics Journal [29], or both [30]. Specific subjects, such as regenerative procedures, have likewise been the object of analysis of the top 100 cited articles [31], and endodontic microbiological research was appraised by analyzing the top 50 most cited articles [32]. 

Currently available journal and author metrics (Journal Impact Factor or h-index, for example) do not always reliably reflect scientific merit and the influence of individual articles; however, the citation count is the index most commonly used to assess their quality. Still, citation counts are highly dependent on academic fields; therefore, calling for field normalization. Citations are moreover slow to reveal the scholarly use of articles and their overall implications: that is, they are time-dependent, underlining the need for time normalization [13]. 

In acknowledging the need for further bibliometric normalization, the National Institutes of Health (NIH) released a new metric operating at the article level: the relative citation ratio (RCR) [33], which is a ratio of rates. The numerator is the article citation rate, calculated as the number of times a paper is cited divided by the number of years since its publication; the denominator is the average citations per year received by NIH-funded publications in the same field contemporaneously. Fields are sampled for each article by means of its co-citation network. While somewhat complicated, the RCR is transparent, and details about its calculation are clearly spelled out in the Hutchins article (2016) [33]. Several bibliometric analyses have found correlations between the RCR and established field-normalized citation indicators [34,35].

Interpreting the RCR is easy. This benchmarking process ensures, by definition, that a paper with an RCR of 1.0 has received the same number of cites/year as the median NIH-funded paper in the same year and field. The RCR is generated for articles indexed in the NIH Open Citation Collection (OCC), their values being freely available at: https://icite.od.nih.gov/analysis (accessed on 15 November 2021). The RCR is held to identify the influence of each article relative to expectations, given the scope of its scientific topic within a specific field [36].

As with all metrics, RCR has limitations [37,38]. It is only calculated from PubMed citations from 1980 onward, so it is not suitable for older or for non-biomedical literature. Being a citation-based metric, RCR is subject to a period of latency, which makes it of limited utility for newly published articles. Another potential limitation is that the RCR uses NIH funding as its benchmark, but it is a standard that can be applied to all articles published in any one of the biomedical fields.

Despite these issues, the RCR is arguably a significant improvement over the most commonly used metrics. It is field-normalized, allowing the comparison of different research areas, which is important since citation patterns can be heterogeneous even in subfields within the same discipline [39]. Furthermore, RCR is time-normalized, and in terms of performance, it is able to identify high-impact papers better shortly after their publication when compared to the non-normalized variants [40], which would be more suitable to identify trends. Although the application of the RCR system is relatively new, and direct comparisons among fields are still developing, the expectations for RCR in bibliometric analysis are great and growing, given its documented usefulness [38]. The RCR was originally designed to assess the scientific impact of NIH-funded research; but it was soon adopted for bibliometric analyses beyond this intended purpose, for instance, to measure the research productivity of scientists or clinicians [41,42,43,44], to evaluate the characteristics of clinical trials [45], or to evaluate the most influential articles [46].

Root canal disinfection is a key part of endodontic and dental research because the success of root canal treatment depends on controlling the infection. While this particular field has produced numerous publications, to the best of our knowledge, the articles of greatest influence—the highest RCRs or top most-cited—have not been identified to date, leaving a research gap. The objective of this bibliometric study was to determine and analyze the 100 articles about root canal disinfection with the highest RCRs from 1990–2019 and to compare them with the top 100 most-cited articles.

## 2. Materials and Methods

### 2.1. Search Strategy, Data Extraction and Citation Metrics

A cross-sectional bibliometric study was undertaken, looking for root canal disinfection with the highest RCRs. PubMed was used as the data source since iCite was designed by the PubMed database. The terms and search strategy were performed by three researchers with experience in endodontics and/or bibliometrics. As the main field of the study includes numerous subfields, it was decided to carry out a broad search strategy using the terms “disinfection” and “root canal” in all fields without limitation of language or type of article. The publications analyzed were limited to 30 years, from 1990 to 2019. The year 2020 was not included because the RCR values of the works published the year prior to the search would have been provisional.

On 27 March 2021, a PubMed search was performed with the following strategy: (Disinfection AND root canal) AND ((“1990/01/01”[Date-Publication]: “2019/12/31”[Date-Publication])), yielding a total of 4294 documents. All the PMID numbers obtained were introduced on the iCite website [47] (https://icite.od.nih.gov/analysis, accessed on 27 March 2021), which recovered 4292 documents (https://icite.od.nih.gov/analysis?search_id=i91628g6ai8tpi4b, accessed on 27 March 2021). The following uploaded PubMed IDs were not found: 29276892 and 28876725. Afterward, the data—publicly available and containing no protected health information—were downloaded for analysis. The available database includes citation data from CrossRef, MedLine, PubMed Central, and Entrez. In addition, reference data were extracted through an automatic learning process applied to open access articles [48].

**Figure 1:**
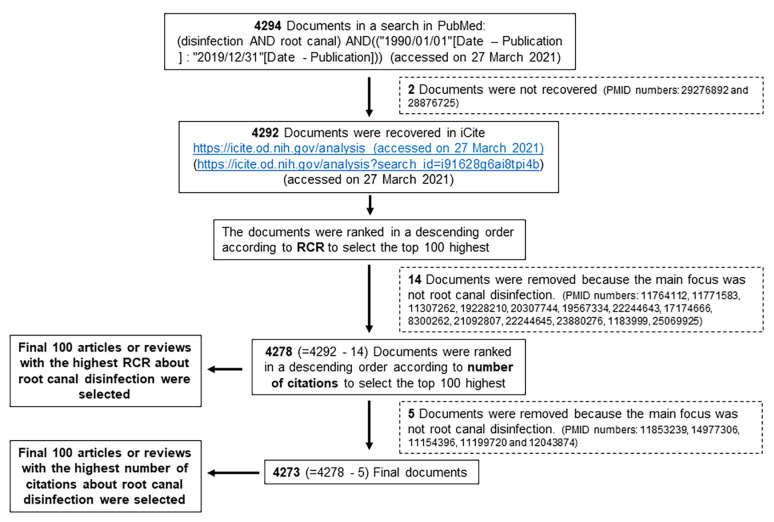
Flow diagram of the different steps taken to select the articles.

The articles were ranked in descending order according to their RCR value, and they were independently assessed by two researchers (PB and CMF-L) to confirm that the main focus was root canal disinfection. A total of 14 documents were eliminated because they did not fit the topic (PMID numbers: 11764112, 11771583, 11307262, 19228210, 20307744, 19567334, 22244643, 17174666, 8300262, 21092807, 22244645, 23880276, 1183999, and 25069925), which allowed us to select the top 100 articles with the highest RCRs. The 4278 documents (=4292 − 14) that remained were then ranked in descending order according to the number of citations, and the same researchers eliminated another five documents for the same reason (PMID numbers: 11853239, 14977306, 11154396, 11199720, and 12043874), after which the top 100 most cited were selected. Figure 1 synthesizes the stages of this process.

All documents were also characterized by the same two researchers, and in the event of disagreement between the two, a unanimous decision had to be made. If more than one article had the same RCR value or number of citations, the article having either more citations or the higher RCR was selected.

Each article was evaluated by metrics of influence, which include RCR, the number of citations, and citations per year. Moreover, they assess the following characteristics: journal, country, and institution of origin of the first author, as well as of the authors and coauthors. The articles were assigned to one of the following subjects or subfields of study: “infection control”, “intracanal medication”, “irrigating solutions”, “irrigation/disinfection techniques”, “regenerative procedures”, “side/interaction effects”, or “smear layer”. They were further characterized by the evidence levels (Els) and study design, whether in vitro (this sector includes studies of parts of dental tissue or sections of the tooth), ex vivo (teeth or complete root), experimental animal, reviews (narrative, systematic, and meta-analysis), clinical observational studies (case report, case series, cross-sectional, single-arm longitudinal study before-after, retrospective case control, and prospective cohort), and clinical experimental studies (quasi-experimental, clinical trial, and randomized clinical trial (RCTs)). 

Vosviewer 1.6.17 software (Centre for Science and Technology Studies, Leiden University, The Netherlands; available at https://www.vosviewer.com, accessed on 7 November 2021) [49] was used to process the data downloaded from Pubmed by constructing a map of bibliographic networks based on keyword co-occurrence from the top 100 articles having the highest RCRs. Keywords with fewer than three occurrences were excluded in order to enhance the clarity of the map. From the top 24 RCR and the top 24 cited articles that were not common to both lists, the network with at least two co-occurring Mesh keywords from the timeline was analyzed. The size of the nodes represented the frequency of the analyzed keyword, so that larger nodes would indicate higher frequency. The thickness of the edges reflects the closeness of the interactions between two nodes, while their colors indicate the cluster to which the keyword belongs. 

### 2.2. Statistical Analysis

The Shapiro–Wilk test was used to test the Gaussian property of RCRs, the number of citations, and citation/year. To compare these metrics among years and decades (1990–1999, 2000–2009, and 2010–2019), and among different Els, study designs (in vitro, ex vivo, review, observational clinical study, or experimental clinical study), and the seven subfields of study, a Kruskal–Wallis test was performed. When the results were statistically significant, a Mann–Whitney test for pair-by-pair comparison was used. This test was likewise used to compare the top 24 RCR against the 24 top-cited articles that were not common to both lists. To study the association between the three time periods and subfield of study, a chi-square test for independence of qualitative variables was applied. The correlation between RCR values and citations in the top 100 RCR articles was evaluated by means of the square of the Pearson linear coefficient. Statistical analysis was performed using SPSS version 26, licensed by the University of Granada. The significance level was set at *p* < 0.05.

## 3. Results

### 3.1. Bibliometrics

Of the 100 root canal disinfection articles identified as having the highest RCRs from 1990–2019, 76 were also identified as among the top 100 most cited, whereas 24 were not on both lists. In Supplementary Materials, Table S1 shows all 124 articles, the corresponding RCR value, number of citations, and the ranking on each list. Table 1 presents the 20 most relevant publications ranked by their RCR, as well as the RCR value and number of citations.

With regard to the top 100 RCR, the highest number of articles published in a single year was seven in the years 2009 and 2011, followed by six articles in 2019. Globally, a significant correlation appeared between RCRs and citations (R^2^ = 71.1%). Bibliometric characteristics are gathered in Table 2, globally and by period (1990–1999, 2000–2009, and 2010–2019). The RCR values are similar among the periods, but the number of citations shows statistically significant differences, with fewer citations in 2010–2019, followed by the period 1990–1999. Citations per year showed similar values in the two latter decades, being significantly higher than in the earliest decade. The analysis of these metrics, by year, shows the same behavior: no differences in RCRs (*p* = 0.386), but differences in citations (*p* = 0.001) and citation per year (*p* < 0.001).

Among the 11 journals represented, the Journal of Endodontics had the largest number of publications, the highest the sum of its articles’ RCRs (weighted RCR) and citations (*n* = 55, 593.69 weighted RCR and 9290 citations), followed by the International Endodontic Journal (*n* = 28, 334.08 weighted RCR and 5006 citations), Oral Surgery, Oral Medicine, Oral Pathology, Oral Radiology and Endodontics (*n* = 5, 56.82 weighted RCR and 913 citations), Endodontics & Dental Traumatology (*n* = 4, 60.40 weighted RCR and 822 citations), and Clinical Oral Investigation (*n* = 2, 15.38 weighted RCR and 29 citations). Six journals participated with only one article: British Dental Journal, Brazilian Dental Journal, Dental Clinics of North America, International Dental Journal, Journal of Dental Research, and Pediatric Dentistry. 

### 3.2. Countries, Institutions and Authors

Based on the institutional address of the first author, 27 countries were represented: the United States with 30 articles, followed by Brazil (*n* = 14) and Canada (*n* = 5). Table 3 displays the countries that contributed to the top 100 RCRs with at least two articles, including their weighted RCR and total citations. 

A total of 60 Institutions contributed to the top 100 RCRs articles. The University of Texas Health Science Center at San Antonio was the best represented, publishing six articles, while second place corresponds to the University of British Columbia, Canada, with five. Table 4 displays the institutions that put out at least two articles, as well as their weighted RCRs and total citations.

A total of 280 authors contributed to the top 100 RCR articles. Number one is M. Haapassalo, with eight articles, followed by two authors from Brazil, each with seven articles. A total of 39 authors published two articles, and 218 contributed with one. 

**Figure 2:**
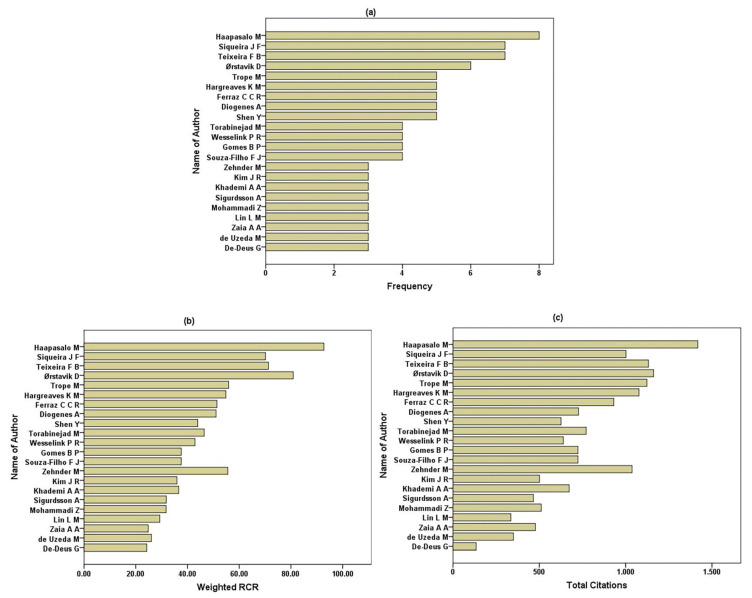
Authors of three or more articles of the 100 root canal disinfection articles with the highest relative citation rates. (**a**) Frequency, (**b**) weighted RCRs (sum of the RCRs for the articles of each author), and (**c**) total citations.

Figure 2 shows the authors who published at least three articles, their frequency (a), weighted RCRs (b), and total citations (c). The mean (standard deviation) of the number of authors/articles was 3.93 (1.89). Four articles were monographs, while 67 articles had between two and four authors.

### 3.3. Study Design, Field of Study, Evidence Level and Keywords

The most frequent study designs were reviews (*n* = 27), nearly all narratives (just one systematic but not from RCTs and no meta-analysis), followed by in vitro (*n* = 25) and ex vivo studies (*n* = 24). Clinical observational studies (*n* = 15) showed different designs: case report (*n* = 5), case series (*n* = 4), case control (*n* = 1), prospective cohort study (*n* = 3), and single-arm longitudinal before–after study (*n* = 2). The least frequent were clinical experimental studies (*n* = 9), which included quasi-experimental (*n* = 2), clinical trial (*n* = 2), and RCTs studies (*n* = 4). This design group included the only experimental animal study. 

Regarding the level of evidence and considering that animal studies, reviews (except systematic review of RCTs), in vitro, and ex vivo studies occupy the bottom of the evidence pyramid [69], the 100 most influential articles in root canal disinfection could be categorized into five Els [70]. The frequency of each level was as follows: EL I (maximum evidence, that is, systematic review of RCTs) (*n* = 0), II (*n* = 4), III (*n* = 9), IV (*n* = 1), and V (*n* = 86). There were no significant differences between Els in the RCR values (*p* = 0.315) but there were in citations (*p* = 0.034).

**Figure 3:**
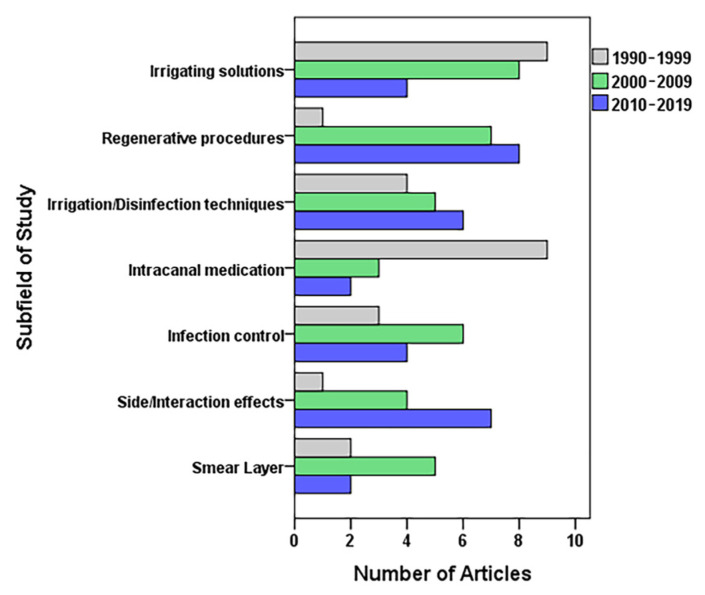
Association between the field of study and time (decade) of publication of the 100 root canal disinfection articles with the highest relative citation rates.

Table 5 indicates the frequency of the articles according to their study design and subfield of study, as well as the weighted RCR, total citations, and the median (interquartile range) of the RCRs and citations. The global comparison of these two variables did not show significant differences for either of the two metrics. Application of the statistical test of independence gave a significant global association (*p* = 0.042) among the three study periods and subfields of study (Figure 3).

**Figure 4:**
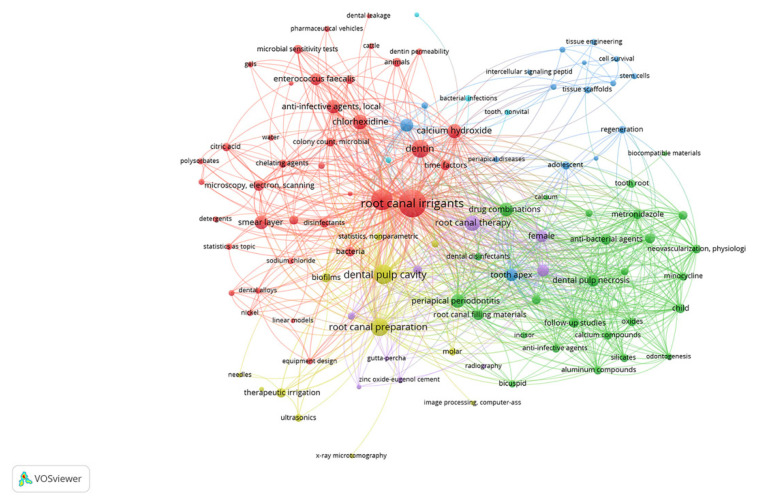
The network of three or more co-occurring keywords of the top 100 RCR articles in root canal disinfection. A total of 106 nodes, six clusters, and a maximum of 800 lines.

Of the 100 main RCR articles, a total of 406 keywords were identified and having three or more co-occurrences, 106 met the limit after excluding “humans” from the list, so that six clusters and 2457 links (total bond strength 6575) were obtained (Figure 4). The most frequently occurring keywords were “root canal irrigants” (*n* = 91), “sodium hypochlorite” (*n* = 52), “dental pulp cavity” (*n* = 44), “root canal preparation”, “dentin” (*n* = 36), “root canal therapy” (*n* = 27), “chlorhexidine” (*n* = 26), “calcium hydroxide” (*n* = 24), “periapical periodontitis” (*n* = 22), “drug combination”, “anti-infective agents local”, and “smear layer” (*n* = 21). Supplementary Materials Table S2 shows all keywords with at least three occurrences.

### 3.4. Top 100 RCRs vs. Top 100 Cited

**Figure 5:**
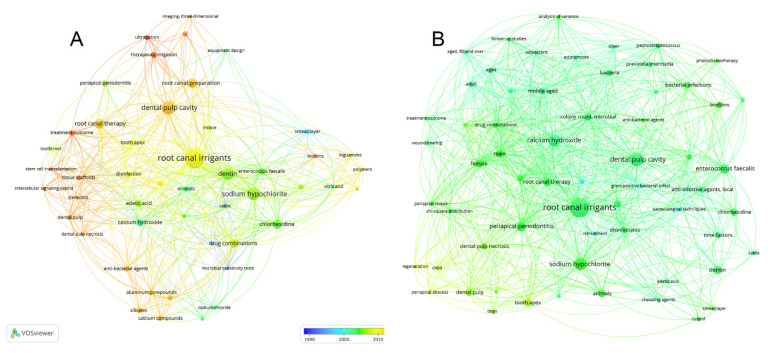
Visualization of Mesh keywords of the highest RCRs and top-cited from the 24 articles not common to both lists having two or more occurrences in the period 1990–2019. The red and orange colors represent the most recent keywords near 2019, while blue and violet zones show the oldest keywords. (**A**). The top 24 RCRs articles: 48 nodes, 5 clusters, and 800 lines. Predominance of green, yellow and orange colors. (**B**). The top 24 cited articles: 62 nodes, 4 clusters, and 800 lines. Predominance of green color.

Upon comparison of the top 100 RCRs and the top 100 most cited, 76 were seen to be common, whereas 24 + 24 articles were not on both lists (Supplementary Materials Table S1). According to the citations of the 100 highest RCRs, 81 are classics with more than 100 cites, while all articles on the most-cited list are classics. Table 6 highlights the bibliometric characteristics of the 24 articles that are not common to both listings. There are significant differences regarding the year of publication, RCRs, citations, and citations per year. From the top 24 RCR articles, a total of 138 Mesh keywords were obtained. Having two or more co-occurrences, 48 met the threshold (excluding “humans”) with 5 clusters, 470 links, and a 777 total link strength. Of the top 24 cited articles, 161 Mesh keywords were obtained. After applying two or more co-occurrences, 62 keywords (excluding “humans”), four clusters with 846 links, and 1315 total link strength were obtained. Figure 5 shows, from both lists, the network keyword timeline with at least two co-occurrences.

## 4. Discussion

Root canal disinfection is an essential aspect of successful endodontics. This study aimed to determine which articles in the field have played the most noteworthy role by identifying and analyzing their characteristics. The RCR is an article-level metric that is field and time-independent; it is strongly correlated with peer-review evaluations and designed to measure the influence of a given publication in its respective research area [33]. It is arguably a significant improvement over other metrics, such a citation counts [38].

The years 1990 to 2019 were chosen because they span the last three decades of work. Since the 1990s, there has been a steady rise in research about endodontic disinfection thanks to various technological developments. In addition, just in the last 30 years, endodontics has been based on biological principles in view of research into endodontic microbiology, intracanal medication, and new materials with greater biocompatibility, among others. The study period analyzed does not take into account the year 2020 (the year prior to the search) because its RCR values are necessarily provisional. By using a 30-year time span parameter, we were able to achieve a finalized list affording a relatively balanced representation of works from the 1990s (*n* = 29), 2000s (*n* = 38), and 2010s (*n* = 33), while demonstrating the progress of root canal disinfection literature.

The 100 articles with the highest RCRs were identified, along with the top 100 most cited, in order to characterize and compare them. It is important to bear in mind that the RCR metric has not been applied in dentistry, a fact that conditions comparisons, as bibliometric studies in endodontics to date have used the number of citations [22,23,24,25,26,27,28,29,30,31,32]

Taking into account the 100 articles selected by the highest RCRs, it is interesting to underline that there are no differences between decades in terms of the RCR metric, which confirms that it is a normalized indicator over time. In contrast, the number of citations shows differences from one period to the next. Lower citations are seen for 2010–2019, as to be expected because citations accumulate over time, hence are said to be time-dependent [71]. The difference between the period 1990–1999 (median = 144) and 2000–2009 (median = 182.5) (Table 2) could be due to the fact that the number of total articles about root canal disinfection in the previous decade evaluated is much lower than that of the period 2000-2009 (537 vs. 1327, data available at http://hdl.handle.net/10481/70539, accessed on 15 November 2021). Given the acceleration in scientific development and output, it is logical that influential older articles would have a much smaller literature readership citing them, as compared to more recent influential articles.

Most of the articles were published in specific journals in this field, especially the Journal of Endodontics (*n* = 55) and the International Endodontic Journal (*n* = 28). Both journals are very well ranked (Q1) in the JCR and are the best-known in endodontics, which may explain why they attract important articles and get more citations. Interestingly, six articles were published in journals not indexed in the JCR, among them Endodontics & Dental Traumatology. In fact, one of its four articles occupies third place in terms of its RCR (*n* = 30.12) and is cited on 393 occasions. In addition, eight articles were published in non-endodontic journals, indicating that it is also a field of interest for dentists in general.

The most influential article of all, with an RCR of 40.46, far above the next in line, is by Zehnder (2006) from the University of Zürich, Switzerland [3], with 774 citations. It takes first place on both lists—top 100 RCRs and top 100 cited—and was the most cited in endodontics during 2000–2009 [30], while also claiming third place in a bibliometric study about citation classics in the Journal of Endodontics [28]. This review article addresses the challenge posed by asepsis in the treatment of root canals, emphasizing the main objective of treatment, the use of optimal disinfection procedures to achieve clinical success and prevent reinfection. It is a fundamental reference article for publications on irrigants in root canal therapy. The second, by Sjögren et al. (1991) from the University of Umeå, Sweden [50], with a 32.40 RCR and 360 citations, is a clinical trial evaluating the antibacterial effect of calcium hydroxide; it demonstrated the efficacy of its use for seven days as intracanal medication, a current recommendation concerning teeth with apical periodontitis or regenerative endodontic therapy. This paper takes 17th place in endodontics [26] and 5th place in an analysis of the top-cited articles in the International Endodontics Journal [29]. The third most influential study is by Orstavik and Haapasalo (1990), from the Scandinavian Institute of Dental Materials, Oslo [51], having an RCR of 30.12 and 393 citations: an in vitro study on bovine infected dentin samples to evaluate the effect of irrigants and intracanal medication. This article, published in Endodontics & Dental Traumatology, is ranked 19th in a 2011 article of the 100 top-cited publications within endodontic journals [26], yet it was not detected when bibliometric studies were focused on specific journals [28,30], which points to the key role of database selection. 

If the Top 100 cited articles are taken into account, the first place, shared with the top 100 RCRs, as mentioned above, is the work of Zehnder (2006) [3]. The second most cited with 440 citations and an RCR of 18.62 is by Stuart et al. (2006) [53], from Wilford Medical Center, Lackland Air Force Base, Texas. It was a review on the role that *Enterococcus faecalis* plays in persistent endodontic infections, but with a clinical focus on how retreatment should be approached. The use of antimicrobial agents and increased size of apical preparation were the most effective methods. This article is ranked 8th in a 2020 article in the period 2000–2009 in two leading journals in endodontics [30], and 19th in an analysis of citation classics in the Journal of Endodontics [28]. The third most cited, with 423 citations, is by Banchs and Trope (2004) [58], from Temple Dental School, Philadelphia. The authors presented a clinical case where a new protocol was applied to treat immature teeth with apical periodontitis. This case report (low level in the pyramid of evidence) meant a very important advance in regenerative endodontics. It is not surprising that it occupies position number five in the period 2000–2009 in endodontics [30] and 14th place in the article about citation classics [28].

The United States gave rise to the greatest number of articles with impact (*n* = 30), a finding consistent with results for dentistry [18,19], endodontics in general [26,28,30], and endodontic microbiology in particular [32]. Notwithstanding, in root canal disinfection, the representation was not so remarkable, due, in part, to the importance of other countries, such as Brazil—with close to 50% of the US output (14 articles). Brazilian research has indeed produced significant output in microbiology within the endodontics field [30,32], their authors and universities appearing on the list of articles having the greatest impact (Table 3 and Figure 2). This has led to a lower relative representation of other European countries that traditionally excelled in endodontics [26,30], or of now-outstanding Asian countries, for instance, in regenerative endodontics [23,31] or pulp therapy [25].

Out of the 60 institutions where the first author was affiliated, 16 pertain to the US, contributing heavily to the highest 100 RCRs in root canal disinfection. The United States is widely accepted as a leader in numerous disciplines, including endodontics [28,30]. The number of US researchers and the relatively well-funded institutions there are viewed as a lighthouse for investigation. Moreover, their authors are more likely to publish in US journals and tend to cite US articles [72]. Among the institutions, the University of Texas Health Science Center at San Antonio was most productive, with six articles. Their works on regenerative procedures are very influential. Next in frequency, with five articles, we find the University of British Columbia in Canada, and very understandably, because this institution harbors the author who published the most articles, M. Haapasalo. With eight articles that amount to a weighted RCR of 85.75 and a total of 1418 citations, he may be considered a top research specialist in this field. Similarly, noteworthy researchers would be J.F. Siqueira and F.B. Teixeira, among others, with seven papers each (Figure 2).

Regarding the subfield of study, we encountered influential articles in all categories, though “irrigating solutions” was most prolific (*n* = 21). It is interesting to note that when we compare the RCRs—as well as citations—there were no significant differences between them (Table 5). This could mean that each one of the subfields of study represents an important area that is well addressed in root canal disinfection studies; a field normalized metric was used [33,36]. Such behavior is likewise observed when the articles were classified according to the study design. While reviews, as anticipated, show a higher median value for RCR (11.19) and citations (184), there were no significant differences among different study designs (Table 5). It is true that reviews tend to be cited more often [73], but at the same time, we are dealing with a collection of articles selected precisely because of their influence calculated in view of citations. 

Overall, we found that few influential studies have designs entailing high scientific evidence: no meta-analysis, only one systematic review, and just four RCTs—despite the fact that systematic reviews and meta-analyses are usually highly cited [73]. Nonetheless, these results should be contemplated in a certain context, namely, considering that the level of evidence in endodontics has its own peculiarities [74], which were taken into account when classifying the articles according to study design. Basic research, through in vitro and ex vivo studies, represents the bulk of papers in endodontics [22]; in our case, nearly half of the articles selected. This means a lesser number of publications in vivo, including RCTs, which in endodontics have moreover shown a tendency to decrease with respect to the growing proportion of reviews [22]. Not surprisingly, then, this study design proved to be the most frequent one (*n* = 27). It is simpler to use—and cite— a review than to work with a series of independent articles. When reviews are of high quality, they simplify work by summing up or critically synthesizing the state-of-art of the research area. Nearly all were narrative (26/27). Even though the trend in dentistry is to publish fewer narratives and more systematic studies [75], within endodontics, the latter are less frequent [76]. These results could be attributed to the predominance of in vitro/ex vivo research studies as opposed to in vivo studies; the systematic ones are generally undertaken in the wake of experimental/observational clinical studies, whereas the narrative/critical ones are founded on basic research. While the methodological quality of systematic reviews is more favorable [77], the common, complementary purpose of the two must be acknowledged. Narrative reviews integrate research from diverse fields, seeking to produce novel insights, and tend to be hypothesis-generating [78]. As mentioned earlier on, the narrative review by Zehnder in 2006 [3] is a solid reference for irrigating solutions in endodontics. 

The in vitro studies (*n* = 25), notwithstanding their low level of evidence (not of quality), is a primary resource for studies of a superior level, and while they may be viewed as far from clinical reality, they are not hampered by confounding factors. In turn, ex vivo studies show a similar frequency (*n* = 24). These designs alter natural conditions as little as possible and may be considered one step closer to in vivo studies. Both are particularly useful when evaluating the elimination of microbial infection by biofilms and for preventing reinfection [74]. 

We encountered an association between the different subfields of study and the period of publication, divided into decades. Most output regarding “intracanal medication” came to light between 1990 and 1999 (nine out of 14), and works on “irrigating solutions” and “smear layer” appeared between 1990 and 2009 (17 out of 21, and 7 out of 9, respectively). The final decade studied, 2010–2019, is more prolific and impacting with regards to disinfection applied to regenerative endodontics (8 of 16). This field has undergone substantial development since the publication of the case report by Banchs and Trope in 2004 [58]. In fact, this paper is ranked 10th in the top 100 RCRs, and 3rd among the top-most cited. Another influential field in recent years would be “irrigation/disinfection techniques”, owing to the technological development of various devices [79], while “side/interaction effects” have grown because of a greater concern for excluding techniques or protocols that might damage adjacent tissues [80].

Bibliometric mapping using VOSviewer (freely available software) proved to be an adequate way to visualize the keywords of the articles. This tool is frequently used in several bibliometric studies published recently in dentistry to perform keyword co-occurrence analysis [24,25,30,32]. The most used keywords were “root canal irrigants”, “sodium hypochlorite”, “dental pulp cavity”, “root canal preparation”, “dentin”, “root canal therapy”, and “chlorhexidine”. Keywords are important elements of any research article involving a bibliographic search and can help researchers when exploring search strategies that lead them to retrieve articles or results considered relevant or pertinent.

It is interesting to note that 76 articles are common to the two lists involved in this study, a number higher than that found for another medical discipline in a similar study, 61/100 [46]. Comparison of the 24 that were not on both lists reveals noteworthy differences in the years of publication (Table 6): a median of 2004 for the top-cited group, 2009 being the most recent year, and a median of 2014 for the top RCRs, 18 of them in the period 2011–2019. This can be visualized in Figure 5, which shows the network keyword timeline with different predominant colors in each list. More yellow-orange nodes—most recent keywords near 2010—in the top 24 RCRs, and green being predominant (older keywords near 2000s) in the 24 top-cited. Only Mesh keywords were selected for this analysis because older articles did not contain author keywords. Citations are time-dependent, so that the older a publication, the more time it has to obtain citations [71]. RCR also needs some time to recount a significant number of citations, and recently published articles have a provisional RCR value [81]. Nonetheless, using this metric enables one to retrieve, as influential, a considerable number of fairly recent articles that would have been lost from the analysis or would have required almetrics to be identified [28]. This stands as an advantage for identifying the most recent trends.

## 5. Strengths and Limitations

This work has strengths and limitations, many of which stem from the metric used. Among the first, we acknowledge the progress that stems from having used a normalized metric versus citation counts without processing. The field-normalized considers the differences in the number of publications and citations for each academic field and subfield. In turn, the normalization of time, although it requires a period for very recent articles being cited (the year 2020 was excluded), allowed us to identify the high-impact and high-quality papers in a short time. In fact, a high number of articles deemed influential that were published in 2019 were obtained. The limitations of this study would be related to the RCR metric. We based our analysis only on articles included in PubMed, ignoring citations beyond the scope of this platform, and as the application of the RCR system is still relatively new, it is difficult to make comparisons. Another potential limitation is that the RCR is derived from benchmarked articles that received NIH funding. In addition, the results must be interpreted, taking into account that the search strategy relied on the two main terms of the topic and that the literature search used in this study was restricted to the period 1990–2019, disregarding older studies.

## 6. Conclusions

Access to the RCR of articles through the web interface, as well as interpretation of the data it provides, proved to be straightforward and user-friendly. The top 100 root canal disinfection articles identified with the highest RCRs between 1990 and 2019 shared 76 with the top 100 cited, and 24 were different in each listing. Despite certain limitations, this metric made it possible to recover, as influential, a high number of relatively recent articles that would have been lost through an analysis of citations. The Journal of Endodontics took the lead regarding articles with the highest RCRs, and the United States followed by Brazil, were the leading countries. Despite advancements in this field, studies entailing high scientific evidence levels are scarce. Most are basic research (in vitro/ex vivo) and narrative reviews. Root canal disinfection is an ever-growing and popular research field, with topics of significance that fluctuate over time. Regenerative procedures, irrigation/disinfection techniques, and side/interaction effects are areas of recent interest. This metric could be the one of choice in future studies, comparing it with other article-level metrics and using a different database in order to address the impact or influence of diverse topics. Research could focus on promising developments in root canal disinfection, such as specific therapeutic strategies based on a greater understanding of the interactions between microorganisms in biofilms or nanoscale materials exerting a broad spectrum of antibacterial activity.

## Figures and Tables

**Table 1:** The 20 most influential root canal disinfection articles ranked according to the highest relative citation rates. 1990–2019.

RCR Rank	Reference	Title	RCR	No. Cites (Cites Rank)
1	Zehnder (2006) [3]	Root canal irrigants.	40.46	774 (1)
2	Sjögren et al. (1991) [50]	The antimicrobial effect of calcium hydroxide as a short-term intracanal dressing.	32.49	360 (6)
3	Orstavik and Haapasalo (1990) [51]	Disinfection by endodontic irrigants and dressings of experimentally infected dentinal tubules.	30.12	393 (4)
4	Sjögren et al. (1997) [52]	Influence of infection at the time of root filling on the outcome of endodontic treatment of teeth with apical periodontitis.	23.38	356 (8)
5	Stuart et al. (2006) [53]	Enterococcus faecalis: its role in root canal treatment failure and current concepts in retreatment.	18.62	440 (2)
6	Nair et al. (2005) [54]	Microbial status of apical root canal system of human mandibular first molars with primary apical periodontitis after “one-visit” endodontic treatment.	18.61	385 (5)
7	Gu et al. (2009) [55]	Review of contemporary irrigant agitation techniques and devices.	18.05	285 (11)
8	Ng et al. (2011) [56]	A prospective study of the factors affecting outcomes of non-surgical root canal treatment: part 1: periapical health.	17.47	256 (13)
9	Jeansonne and White (1994) [57]	A comparison of 2.0% chlorhexidine gluconate and 5.25% sodium hypochlorite as antimicrobial endodontic irrigants.	17.46	207 (20)
10	Banchs and Trope (2004) [58]	Revascularization of immature permanent teeth with apical periodontitis: new treatment protocol?	16.76	423 (3)
11	Siqueira and Lopes (1999) [59]	Mechanisms of antimicrobial activity of calcium hydroxide: a critical review.	16.75	331 (9)
12	Safavi et al. (1990) [60]	Root canal dentinal tubule disinfection.	16.18	152 (49)
13	Torabinejad et al. (2003) [61]	A new solution for the removal of the smear layer.	15.41	289 (10)
14	Nerwich et al. (1993) [62]	pH changes in root dentin over a 4-weekperiod following root canal dressing with calcium hydroxide.	15.29	159 (42)
15	van der Sluis et al. (2007) [63]	Passive ultrasonic irrigation of the root canal: a review of the literature.	14.7	280 (12)
16	Murray et al. (2007) [64]	Regenerative endodontics: a review of current status and a call for action.	13.95	357 (7)
17	Ruparel et al. (2012) [65]	Direct effect of intracanal medicaments on survival of stem cells of the apical papilla	13.8	207 (21)
18	Shuping et al. (2000) [66]	Reduction of intracanal bacteria using nickel-titanium rotary instrumentation and various medications.	13.49	245 (15)
19	Torabinejad et al. (2002) [67]	Clinical implications of the smear layer in endodontics: a review.	13.37	245 (16)
20	Mohammadi and Dummer (2011) [68]	Properties and applications of calcium hydroxide in endodontics and dental traumatology.	13.17	195 (27)

**Table 2:** Metrics of the top 100 most influential root canal disinfection articles, with the highest relative citation rates (RCRs): 1990–2019.

	1990–2019 *n* = 100	1990–1999 *n* = 29	2000–2009 *n* = 38	2010–2019 *n* = 33	Comparison *p*-Value *
RCRs ^a^	9.34 (4.24)	9.75 (5.23)	9.59 (5.63)	9.28 (2.46)	0.280
Min–Max	7.23–40.46	7.55–32.49	7.27–40.46	7.23–17.47	
Weighted RCR	1111.73	355.93	436.92	318.88	
Cites ^a^	148 (84.25)	144 (53) ^1^	182.5(105) ^2^	108 (97) ^3^	<0.001
Min-Max	14–774	75–393	99–774	14–256	
Total citations	16870	4838	8354	3678	
Citations/year ^a^	11 (8.18)	5.62 (3.79) ^1^	11.28 (6.03) ^2^	13.77 (6.02) ^2^	<0.001

^a^ Median (interquartile range). Min–Max: Minimum and Maximum values. Weighted RCR: sum of the RCRs for the articles in the group. * Kruskal–Wallis test, previously the Shapiro–Wilks test showed no normality. Pair-by-pair comparison by Mann–Whitney test. Read horizontally, the same superscript number indicates a non-significant difference.

**Table 3:** Countries that contributed with at least two articles to the top 100 most influential root canal disinfection articles with the highest relative citation rates (RCRs): 1990–2019.

Rank	Country *	No of Articles	Weighted RCR	Total Citations
1	United States	30	329.36	5448
2	Brazil	14	131.23	1857
3	Canada	5	43.94	642
4	Switzerland	4	74.20	1422
5	United Kingdom	4	45.99	768
6	Iran	4	41.55	596
7	The Netherlands	4	41.09	495
8	Germany	4	38.92	647
9	Italy	4	35.08	405
10	Norway	3	48.98	612
11	Turkey	3	34.45	524
12	Sweden	2	55.87	716
13	China	2	27.28	461
14	Korea	2	18.67	343
15	Japan	2	17.87	281
16	India	2	15.99	272

Weighted RCR: sum of the RCRs for the articles of each country. * A total of 27 countries contributed to the top 100 RCR articles. Countries with the same number of articles were ordered according to their highest weighted RCR.

**Table 4:** Institutions that contributed at least two articles to the top 100 most influential root canal disinfection articles with the highest relative citation rates (RCRs): 1990–2019.

Rank	Institutions * (Countries)	No. of Articles	Weighted RCR	Total Citations
1	University of Texas Health Science Center at San Antonio (United States)	6	63.73	962
2	University of British Columbia (Canada)	5	43.94	642
3	University of Zürich (Switzerland)	4	55.59	1422
4	Loma Linda University (United States)	4	46.45	822
5	Academic Centre for Dentistry, Amsterdam (The Netherlands)	4	41.09	513
6	University of North Carolina (United States)	4	39.15	700
7	University of Campinas (Brazil)	4	37.58	630
8	Federal University of Rio de Janeiro (Brazil)	3	26.06	413
9	University of Umeå (Sweden)	2	55.87	716
10	University of Texas Health Science Center, Houston (United States)	2	28.70	365
11	University College London (United Kingdom)	2	27.96	404
12	University of Connecticut Health Center (United States)	2	24.50	274
13	Hamedan University of Medical Sciences (Iran)	2	24.36	380
14	Estácio de Sá University, Rio de Janeiro (Brazil)	2	23.95	449
15	Private Practice (New Zealand, Italy)	2	22.99	226
16	University of Göttingen (Germany)	2	22.80	414
17	Ege University (Turkey)	2	21.81	325
18	University of Oregon Health Sciences Center (United States)	2	19.75	268
19	University of Oslo (Norway)	2	18.86	219
20	Yonsei University (Korea)	2	18.67	343
21	University of Siena (Italy)	2	16.25	248
22	University of Regensburg (Germany)	2	16.12	233

Weighted RCR: sum of the RCRs for the articles of each institution. * A total of 60 institutions contributed to the top 100 RCR articles. Institutions with the same number of articles were ordered according to their highest weighted RCR.

**Table 5:** Study design and field of study of the top 100 most influential articles in root canal disinfection by the highest RCRs. 1990–2019. Weighted RCR, total citations. Median (interquartile range).

Study Design	*n*	RCR		Citations	
		Weighted	Median (IR)	Total	Median (IR)
In vitro	25	333.94	9.9 (3.51)	5305	131 (91.5)
Ex vivo	24	262.06	8.73 (2.6)	3773	129 (48.75)
Review	27	230.99	11.19 (5.09)	3223	184 (143)
Clinical observational	15	173.66	9.83 (5.03)	2998	170 (97)
Clinical experimental	9	111.08	9.01 (5.94)	1571	135 (190.5)
Comparison *p*-value			0.573 *		0.270 *
**Field of Study**					
Irrigating solutions	21	261.99	9.83 (4.07)	4005	148 (82.5)
Regenerative procedures	16	157.23	9.19 (1.99)	2566	149 (87.75)
Irrigation/disinfection techniques	15	147.59	8.07 (0.96)	1807	117 (143)
Intracanal medication	14	174.54	9.85 (6.65)	2630	168.5 (53.75)
Infection control	13	162.95	10.49 (9.58)	2578	181 (194)
Side/interaction effects	12	112.04	10.01 (3.25)	1806	146.5 (68)
Smear layer	9	92.39	8.7 (5.61)	1478	141 (114)
Comparison *p*-value			0.136 *		0.115 *

Weighted RCR: sum of the RCRs for the articles of each study design or field of study. * Global comparison by Kruskal–Wallis test. Data not following normal distribution determined by Shapiro–Wilk test.

**Table 6:** Comparison of characteristics of root canal disinfection articles with the highest RCRs and top-cited. The 24 articles not common to both lists 1990–2019.

		Top RCR *n* = 24	Top Cited *n* = 24	Comparison *p*-Value *
Year ^a^		2014 (27)	2004 (14)	0.001
	Min–Max	1992–2019	1995–2009	
RCRs ^a^		8.37 (3.57)	6.63 (2.17)	<0.001
	Min–Max	7.23–10.80	5.04–7.21	
	Weighted RCR	205.78	156.60	
Cites ^a^		84.5 (95)	130 (52)	<0.001
	Min–Max	14–109	111–163	
	Total citations	1718	3165	
Citations/year ^a^		11 (13.74)	7.59 (7.21)	0.042

^a^ Median (interquartile range). Min–Max: Minimum and Maximum values. Weighted RCR: sum of the RCRs for the articles in the group. * Mann–Whitney test, previously the Shapiro–Wilk test, showed no normality.

## Data Availability

Data associated with this article are available on the Digibug Project page at http://hdl.handle.net/10481/70539 (accessed on 15 November 2021).

## Supplementary Materials

The following are available online at https://www.mdpi.com/article/10.3390/antibiotics10111412/s1, Table S1: The top 100 most influential root canal disinfection articles ranked by the highest relative citation rates and top 100 most cited. 1990–2019, Table S2: Keywords with at least three co-occurrences among the top 100 RCR articles in root canal disinfection.
