# Supplementary material for: Root Canal Disinfection Articles with the Highest Relative Citation Ratios. A Bibliometric Analysis from 1990 to 2019
Source: Antibiotics (Basel). 2021 Nov 18;10(11):1412. doi: 10.3390/antibiotics10111412 (PMC8614753; doi:10.3390/antibiotics10111412)
Supplement: Supplementary file 1 [file antibiotics-10-01412-s001.zip › Supplementary Materials Table S1.pdf]

Table S1. The top 100 most influential root canal disinfection articles ranked by the highest relative citation rates and top 100 most cited. 1990-2019.

RCR RANK	Nº cites RANK	References	RCR	No. cites
1	1	Zehnder, M. Root canal irrigants. *J Endod*. **2006**, *32*, 389-398.	40.46	774
2	6	Sjögren, U.; Figdor, D.; Spångberg, L.; Sundqvist, G. The antimicrobial effect of calcium hydroxide as a short-term intracanal dressing. *Int Endod J*. **1991**, *24,*119-25.	32.49	360
3	4	Orstavik, D.; Haapasalo, M. Disinfection by endodontic irrigants and dressings of experimentally infected dentinal tubules. *Endod Dent Traumatol*. **1990,** *6*, 142-9.	30.12	393
4	8	Sjögren, U.; Figdor, D.; Persson, S.; Sundqvist, G. Influence of infection at the time of root filling on the outcome of endodontic treatment of teeth with apical periodontitis. *Int Endod J*. **1997**, *30*, 297-306.	23.38	356
5	2	Stuart, C.H.; Schwartz, S.A.; Beeson, T.J.; Owatz, C.B. Enterococcus faecalis: its role in root canal treatment failure and current concepts in retreatment. *J Endod*. **2006**, *32*, 93-8.	18.62	440
6	5	Nair, P.N.; Henry, S.; Cano, V.; Vera, J. Microbial status of apical root canal system of human mandibular first molars with primary apical periodontitis after "one-visit" endodontic treatment. *Oral Surg Oral Med Oral Pathol Oral Radiol Endod*. **2005**, *99*, 231-52.	18.61	385
7	11	Gu, L.S.; Kim, J.R.; Ling, J.; Choi, K.K.; Pashley, D.H.; Tay, F.R. Review of contemporary irrigant agitation techniques and devices. *J Endod*. **2009**, *35*, 791-804.	18.05	285
8	13	Ng, Y.L.; Mann, V.; Gulabivala, K. A prospective study of the factors affecting outcomes of non surgical root canal treatment: part 1: periapical health. *Int Endod J*. **2011**, *44*, 583-609.	17.47	256
9	20	Jeansonne MJ, White RR. A comparison of 2.0% chlorhexidine gluconate and 5.25% sodium hypochlorite as antimicrobial endodontic irrigants. *J Endod*. **1994**, *20*, 276-8.	17.46	207
10	3	Banchs, F.; Trope, M. Revascularization of immature permanent teeth with apical periodontitis: new treatment protocol? *J Endod*. **2004**, *30*, 196-200.	16.76	423
11	9	Siqueira, J.F.; Lopes, H.P. Mechanisms of antimicrobial activity of calcium hydroxide: a critical review. *Int Endod J*. **1999**, *32*, 361-9.	16.75	331
12	49	Safavi, K.E.; Spangberg, L.S.; Langeland, K. Root canal dentinal tubule disinfection. *J Endod*. **1990**, *16*, 207-10.	16.18	152
13	10	Torabinejad, M.; Khademi, A.A.; Babagoli, J.; Cho, Y.; Johnson, W.B.; Bozhilov, K.; Kim, J.; Shabahang, S. A new solution for the removal of the smear layer. *J Endod*. **2003***;* *29*, 170-5.	15.41	289
14	42	Nerwich, A.; Figdor, D.; Messer, H.H. pH changes in root dentin over a 4-weekperiod following root canal dressing with calcium hydroxide. *J Endod*. **1993**, *19*, 302-6.	15.29	159
15	12	van der Sluis, L.W.; Versluis, M.; Wu, M.K.; Wesselink, P.R. Passive ultrasonicirrigation of the root canal: a review of the literature. *Int Endod J*. **2007**, *40*, 415-26.	14.7	280
16	7	Murray, P.E.; Garcia-Godoy, F.; Hargreaves, K.M. Regenerative endodontics: a review of current status and a call for action. *J Endod*. **2007**, *33*, 377-90.	13.95	357
17	21	Ruparel, N.B.; Teixeira, F.B.; Ferraz, C.C.; Diogenes, A. Direct effect of intracanal medicaments on survival of stem cells of the apical papilla. *J Endod*. **2012**, *38*, 1372-5.	13.8	207
18	15	Shuping, G.B.; Orstavik, D.; Sigurdsson, A.; Trope, M. Reduction of intracanal bacteria using nickel-titanium rotary instrumentation and various medications. *J Endod*. **2000**, *26*, 51-5.	13.49	245
19	16	Torabinejad, M.; Handysides. R.; Khademi, A.A.; Bakland, L.K. Clinical implications of the smear layer in Endodontics: a review. *Oral Surg Oral Med Oral Pathol Oral Radiol Endod*. **2002**, *94*, 658-66.	13.37	245

20	27	Mohammadi, Z.; Dummer, P.M. Properties and applications of calcium hydroxide in endodontics and dental traumatology. *Int Endod J*. **2011**, *44*, 697-730.	13.17	195
21	22	Violich, D.R.; Chandler, N.P. The smear layer in endodontics - a review. Int *Endod J*. **2010**, *43,* 2-15.	13.15	204
22	14	Gomes, B.P.; Ferraz, C.C.; Vianna, M.E.; Berber, V.B.; Teixeira, F.B.; Souza-Filho, F.J. In vitro antimicrobial activity of several concentrations of sodium hypochlorite and chlorhexidine gluconate in the elimination of Enterococcus faecalis. *Int Endod J*. **2001**, *34*, 424-8.	12.72	247
23	17	Bose, R.; Nummikoski, P.; Hargreaves, K. A retrospective evaluation of radiographic outcomes in immature teeth with necrotic root canal systems treated with regenerative endodontic procedures. *J Endod*. **2009**, *35*, 1343-9.	12.68	235
24	18	Calt, S.; Serper, A. Time-dependent effects of EDTA on dentin structures. *J Endod*. **2002**, *28*, 17-9.	12.64	232
25	25	Haapasalo, M.; Shen, Y.; Qian, W.; Gao, Y. Irrigation in endodontics. *Dent Clin North Am*. **2010**, *54*, 291-312.	12.49	199
26	73	Baumgartner, J.C.; Cuenin, P.R. Efficacy of several concentrations of sodium hypochlorite for root canal irrigation. *J Endod*. **1992**, *18*, 605-12.	12.14	131
27	19	Hülsmann, M.; Heckendorff, M.; Lennon, A. Chelating agents in root canal treatment: mode of action and indications for their use. *Int Endod J*. **2003**, *36*, 810-30.	11.53	221
28	60	Sen, B.H.; Wesselink, P.R.; Türkün, M. The smear layer: a phenomenon in root canal therapy. *Int Endod J*. **1995**, *28*, 141-8.	11.32	144
29	28	Hülsmann, M.; Hahn, W. Complications during root canal irrigation-literature review and case reports. *Int Endod J*. **2000**, *33*, 186-93.	11.27	193
30	43	White, R.R.; Hays, G.L.; Janer, L.R. Residual antimicrobial activity after canal irrigation with chlorhexidine. *J Endod*. **1997**, *23*, 229-31.	11.24	158
31	29	Mohammadi, Z.; Abbott, P.V. The properties and applications of chlorhexidine inendodontics. *Int Endod J*. **2009**, *42*, 288-302.	11.19	185
32	45	Chen, M.Y.; Chen, K.L.; Chen, C.A.; Tayebaty, F.; Rosenberg, P.A.; Lin, L.M. Responses of immature permanent teeth with infected necrotic pulp tissue and apical periodontitis/abscess to revascularization procedures. *Int Endod J*. **2012**, *45*, 294-305.	10.87	157
33	-	Swimberghe, R.C.D.; Coenye, T.; De Moor, R.J.G.; Meire, M.A. Biofilm model systems for root canal disinfection: a literature review. *Int Endod J*. **2019**, *52*, 604-628.	10.8	26
34	39	Tay, F.R.; Gu, L.S.; Schoeffel, G.J.; Wimmer, C.; Susin, L.; Zhang, K.; Arun, S.N.; Kim, J.; Looney, S.W.; Pashley, D.H. Effect of vapor lock on root canal debridement by using aside-vented needle for positive-pressure irrigant delivery. *J Endod*. **2010**, *36*, 745-50.	10.56	165
35	31	Sim, T.P.; Knowles, J.C.; Ng, Y.L.; Shelton, J.; Gulabivala, K. Effect of sodium hypochlorite on mechanical properties of dentine and tooth surface strain. *Int Endod J*. **2001**, *34*, 120-32.	10.49	181
36	54	Sen, B.H.; Piskin, B.; Demirci, T. Observation of bacteria and fungi in infected root canals and dentinal tubules by SEM. *Endod Dent Traumatol*. **1995**, *11*, 6-9.	10.49	148
37	82	Martin, D.E.; De Almeida, J.F.; Henry, M.A.; Khaing, Z.Z.; Schmidt, C.E.; Teixeira, F.B.; Diogenes, A. Concentration-dependent effect of sodium hypochlorite on stem cells of apical papilla survival and differentiation. *J Endod*. **2014**, *40*, 51-5.	10.28	123
38	34	Kim, J.H.; Kim, Y.; Shin, S.J.; Park, J.W.; Jung, I.Y. Tooth discoloration of immature permanent incisor associated with triple antibiotic therapy: a case report. *J Endod*. **2010**, *36*, 1086-91.	10.21	173
39	23	Haapasalo, H.K.; Sirén, E.K.; Waltimo, T.M.; Ørstavik, D.; Haapasalo, M.P. Inactivation of local root canal medicaments by dentine: an in vitro study. *Int Endod J*. **2000**, *33*, 126-31.	10.01	203
40	-	Vahdaty, A.; Pitt Ford, T.R.; Wilson, R.F. Efficacy of chlorhexidine in disinfecting dentinal tubules in vitro. *Endod Dent Traumatol*. **1993**, 9, 243-8.	9.96	99

41	24	Gomes, B.P.; Souza, S.F.; Ferraz, C.C.; Teixeira, F.B.; Zaia, A.A.; Valdrighi, L.; Souza-Filho, F.J. Effectiveness of 2% chlorhexidine gel and calcium hydroxide against Enterococcus faecalis in bovine root dentine in vitro. *Int Endod J*. **2003**, *36*, 267-75.	9.96	200
42	51	Hargreaves, K.M.; Diogenes, A.; Teixeira, F.B. Treatment options: biological basis of regenerative endodontic procedures. *J Endod*. **2013**, *39*, S30-43.	9.92	149
43	-	Plotino, G.; Grande, N.M.; Mercade, M. Photodynamic therapy in endodontics. *Int Endod J*. **2019**, *52*, 760-774.	9.84	22
44	52	Nosrat, A.; Seifi, A.; Asgary, S. Regenerative endodontic treatment (revascularization) for necrotic immature permanent molars: a review and reportof two cases with a new biomaterial. *J Endod*. **2011**, *37*, 562-7.	9.83	149
45	-	Ohara, P.; Torabinejad, M.; Kettering, J.D. Antibacterial effects of various endodontic irrigants on selected anaerobic bacteria. *Endod Dent Traumatol*. **1993**, *9*, 95-100.	9.83	98
46	-	Levy, G. Cleaning and shaping the root canal with a Nd:YAG laser beam: a comparative study. *J Endod*. **1992**, *18*, 123-7.	9.83	85
47	38	Trevino, E.G.; Patwardhan, A.N.; Henry, M.A.; Perry, G.; Dybdal-Hargreaves, N.; Hargreaves, K.M.; Diogenes, A. Effect of irrigants on the survival of human stem cells of the apical papilla in a platelet-rich plasma scaffold in human root tips. *J Endod*. **2011**, *37*, 1109-15.	9.82	166
48	40	Sato, I.; Ando-Kurihara, N.; Kota, K.; Iwaku, M.; Hoshino, E. Sterilization of infected root-canal dentine by topical application of a mixture of ciprofloxacin, metronidazole and minocycline in situ. *Int Endod J*. **1996**, *29*, 118-24.	9.75	164
49	-	Căpută, P.E.; Retsas, A.; Kuijk, L.; Chávez de Paz, L.E.; Boutsioukis, C. Ultrasonic irrigant activation during root canal treatment: a systematic review. *J Endod*. **2019**, *45*, 31-44.e13.	9.52	18
50	70	Trope, M.; Delano, E.O.; Orstavik, D. Endodontic treatment of teeth with apical periodontitis: single vs. multivisit treatment. *J Endod*. **1999**, *25*, 345-50.	9.38	132
51	80	Vera, J.; Siqueira, J.F. Jr.; Ricucci, D.; Loghin, S.; Fernández, N.; Flores, B.; Cruz, A.G. One-versus two-visit endodontic treatment of teeth with apical periodontitis: a histobacteriologic study. *J Endod*. **2012**, *38*, 1040-52.	9.31	124
52	55	Leonardo, M.R.; Tanomaru Filho, M.; Silva, L.A.; Nelson Filho, P.; Bonifácio, K.C.; Ito, I.Y. In vivo antimicrobial activity of 2% chlorhexidine used as a root canal irrigating solution. *J Endod*. **1999**, *25*, 167-71.	9.29	148
53	-	Kim, S.G.; Malek, M.; Sigurdsson, A.; Lin, L.M.; Kahler, B. Regenerative endodontics: a comprehensive review. *Int Endod J*. **2018**, *51*, 1367-1388.	9.28	49
54	33	Ding, R.Y.; Cheung, G.S.; Chen, J.; Yin, X.Z.; Wang, Q.Q.; Zhang, C.F. Pulp revascularization of immature teeth with apical periodontitis: a clinical study. *J Endod*. **2009**, *35*, 745-9.	9.23	176
55	92	Siqueira, J.F. Jr.; de Uzeda, M. Intracanal medicaments: evaluation of the antibacterial effects of chlorhexidine, metronidazole, and calcium hydroxide associated with three vehicles. *J Endod*. **1997**, *23*, 167-9.	9.2	118
56	32	Siqueira, J.F. Jr.; Rôças, I.N.; Favieri, A.; Lima, K.C. Chemomechanical reduction of the bacterial population in the root canal after instrumentation and irrigation with 1%, 2.5%, and 5.25% sodium hypochlorite. *J Endod*. **2000**, *26*, 331-4.	9.2	181
57	76	Martin, G.; Ricucci, D.; Gibbs, J.L.; Lin, L.M. Histological findings of revascularized/revitalized immature permanent molar with apical periodontitis using platelet-rich plasma. *J Endod*. **2013**, *39*, 138-44.	9.16	129
58	36	Card, S.J.; Sigurdsson, A.; Orstavik, D.; Trope, M. The effectiveness of increased apical enlargement in reducing intracanal bacteria. *J Endod*. **2002**, *28*, 779-83.	9.01	172
59	66	Berutti, E.; Marini, R.; Angeretti, A. Penetration ability of different irrigants into dentinal tubules. *J Endod*. **1997**, *23*, 725-7.	8.99	135
60	-	Versiani, M.A.; Leoni, G.B.; Steier, L.; De-Deus, G.; Tassani, S.; Pécora, J.D.; de Sousa-Neto, M.D. Micro-computed tomography study of oval-shaped canals	8.88	105

		prepared with the self-adjusting file, Reciproc, WaveOne, and ProTaper universal systems. *J Endod*. **2013**, *39*, 1060-6.		
61	-	Zandi, H.; Petronijevic, N.; Mdala, I.; Kristoffersen, A.K.; Enersen, M.; Rôças, I.N.; Siqueira, J.F. Jr.; Ørstavik, D. Outcome of Endodontic retreatment using 2 root canal irrigants and influence of infection on healing as determined by a molecular method: a randomized clinical trial. *J Endod*. **2019**, *45*, 1089-1098.e5.	8.85	16
62	-	Haapasalo, M.; Shen, Y.; Wang, Z.; Gao, Y. Irrigation in Endodontics. *Br Dent J*. **2014**, *216*, 299-303.	8.81	94
63	-	Garberoglio, R.; Becce, C. Smear layer removal by root canal irrigants. A comparative scanning electron microscopic study. *Oral Surg Oral Med Oral Pathol*. **1994**, *78*, 359-67.	8.7	95
64	26	Peciuliene, V.; Reynaud, A.H.; Balciuniene, I.; Haapasalo, M. Isolation of yeasts andenteric bacteria in root-filled teeth with chronic apical periodontitis. *Int Endod J*. **2001**, *34*, 429-34.	8.68	196
65	77	Siqueira, J.F. Jr.; Batista, M.M.; Fraga, R.C.; de Uzeda, M. Antibacterial effects of endodontic irrigants on black-pigmented gram-negative anaerobes and facultative bacteria. *J Endod*. **1998**, *24*, 414-6.	8.61	129
66	98	Wu, M.K.; Wesselink, P.R. Efficacy of three techniques in cleaning the apical portion of curved root canals. *Oral Surg Oral Med Oral Pathol Oral Radiol Endod*.**1995**, *79*, 492-6.	8.59	113
67	-	Galler, K.M.; Buchalla, W.; Hiller, K.A.; Federlin, M.; Eidt, A.; Schiefersteiner, M.; Schmalz, G. Influence of root canal disinfectants on growth factor release fromdentin. *J Endod*. **2015**, *41*, 363-8.	8.5	92
68	37	Jung, I.Y.; Lee, S.J.; Hargreaves, K.M. Biologically based treatment of immature permanent teeth with pulpal necrosis: a case series. *J Endod*. **2008**, *34*, 876-87.	8.46	170
69	83	Kuruvilla, J.R.; Kamath, M.P. Antimicrobial activity of 2.5% sodium hypochlorite and 0.2% chlorhexidine gluconate separately and combined, as endodontic irrigants. *J Endod*. **1998**, *24*, 472-6.	8.37	123
70	85	Safavi, K.E.; Nichols, F.C. Effect of calcium hydroxide on bacterial lipopolysaccharide. *J Endod*. **1993**, *19*, 76-8.	8.32	122
71	-	Kontakiotis, E.G.; Filippatos, C.G.; Tzanetakis, G.N.; Agrafioti, A. Regenerative endodontic therapy: a data analysis of clinical protocols. *J Endod*. **2015**, *41*, 146-54.	8.28	84
72	-	Peters, L.B.; Wesselink, P.R.; Moorer, W.R. The fate and the role of bacteria left in root dentinal tubules. *Int Endod J*. **1995**, *28*, 95-9.	8.28	102
73	-	Siqueira, J.F. Jr.; de Uzeda, M. Disinfection by ;alcium hydroxide pastes of dentinal tubules infected with two obligate and one facultative anaerobic bacteria. *J Endod*. **1996,** *22*, 674-6.	8.23	103
74	-	Silva, E.J.N.L.; Carvalho, C.R.; Belladonna, F.G.; Prado, M.C.; Lopes, R.T.; De-Deus, G., Moreira, E.J.L. Micro-CT evaluation of different final irrigation protocols on the removal of hard-tissue debris from isthmus-containing mesial root of mandibular molars. *Clin Oral Investig*. **2019**, *23*, 681-687.	8.14	15
75	94	Takeda, F.H.; Harashima, T.; Kimura, Y.; Matsumoto, K. A comparative study of the removal of smear layer by three endodontic irrigants and two types of laser. *Int Endod J*. **1999**, *32*, 32-9.	8.12	117
76	30	Estrela, C.; Estrela, C.R.; Barbin, E.L.; Spanó, J.C.; Marchesan, M.A.; Pécora, J.D. Mechanism of action of sodium hypochlorite. *Braz Dent J*. **2002**, *13*, 113-7.	8.11	184
77	-	Wigler, R.; Kaufman, A.Y.; Lin, S.; Steinbock, N.; Hazan-Molina, H.; Torneck, C.D. Revascularization: a treatment for permanent teeth with necrotic pulp and incomplete root development. *J Endod*. **2013**, *39*, 319-26.	8.1	109
78	74	Fava, L.R.; Saunders, W.P. Calcium hydroxide pastes: classification and clinical indications. *Int Endod J*. **1999**, *32*, 257-82.	8.07	131
79	91	Desai, P.; Himel, V. Comparative safety of various intracanal irrigation systems. *J Endod*. **2009**, *35*, 545-9.	7.95	119

80	58	Reynolds, K.; Johnson, J.D.; Cohenca, N. Pulp revascularization of necrotic bilateral bicuspids using a modified novel technique to eliminate potential coronal discolouration: a case report. *Int Endod J.* **2009**, *42*, 84-92.	7.94	145
81	64	Torabinejad, M.; Cho, Y.; Khademi, A.A.; Bakland, L.K.; Shabahang, S. The effect of various concentrations of sodium hypochlorite on the ability of MTAD to remove the smear layer. *J Endod.* **2003**, *29*, 233-9.	7.84	139
82	86	Lottanti, S.; Gautschi, H.; Sener, B.; Zehnder, M. Effects of ethylenediaminetetraacetic, etidronic and peracetic acid irrigation on human root dentine and the smear layer. *Int Endod J.* **2009**, *42*, 335-43.	7.83	122
83	79	Shen, Y.; Stojicic, S.; Haapasalo, M. Antimicrobial efficacy of chlorhexidine against bacteria in biofilms at different stages of development. *J Endod.* **2011**, *37*, 657-61.	7.82	126
84	-	Camilleri, J. Color stability of white mineral trioxide aggregate in contact with hypochlorite solution. *J Endod.* **2014**, *40*, 436-40.	7.79	77
85	53	Shah, N.; Logani, A.; Bhaskar, U.; Aggarwal, V. Efficacy of revascularization to induce apexification/apexogensis in infected, nonvital, immature teeth: a pilot clinical study. *J Endod.* **2008**, *34*, 919-25; Discussion 1157.	7.62	149
86	61	Galler, K.M.; D'Souza, R.N.; Federlin, M.; Cavender, A.C.; Hartgerink, J.D.; Hecker, S.; Schmalz, G. Dentin conditioning codetermines cell fate in regenerative endodontics. *J Endod.* **2011**, *37*, 1536-41.	7.62	141
87	65	Nielsen, B.A.; Craig Baumgartner, J. Comparison of the EndoVac system to need irrigation of root canals. *J Endod.* **2007**, *33*, 611-5.	7.61	137
88	48	Vianna, M.E.; Gomes, B.P.; Berber, V.B.; Zaia, A.A.; Ferraz, C.C.; de Souza-Filho, F.J. In vitro evaluation of the antimicrobial activity of chlorhexidine and sodium hypochlorite. *Oral Surg Oral Med Oral Pathol Oral Radiol Endod.* **2004**, *97*, 79-84.	7.55	153
89	-	Chong, B.S.; Pitt Ford, T.R. The role of intracanal medication in root canal treatment. *Int Endod J.* **1992**, *25*, 97-106.	7.55	75
90	-	Stojicic, S., Shen, Y.; Qian, W.; Johnson, B.; Haapasalo, M. Antibacterial and smear layer removal ability of a novel irrigant, QMiX. *Int Endod J.* **2012**, *45*, 363-71.	7.47	99
91	67	Gutarts, R.; Nusstein, J.; Reader, A.; Beck, M. In vivo debridement efficacy of ultrasonic irrigation following hand-rotary instrumentation in human mandibular molars. *J Endod.* **2005**, *31*, 166-70.	7.43	135
92	75	Mohammadi, Z. Sodium hypochlorite in Endodontics: an update review. *Int Dent J.* **2008**, *58*, 329-41.	7.36	131
93	81	Ferraz, C.C.; Gomes, B.P.; Zaia, A.A.; Teixeira, F.B.; Souza-Filho, F.J. In vitro assessment of the antimicrobial action and the mechanical ability of chlorhexidine gel as an endodontic irrigant. *J Endod.* **2001**, *27*, 452-5.	7.35	124
94	-	Ma, J.; Wang, Z.; Shen, Y.; Haapasalo, M. A new noninvasive model to study the effectiveness of dentin disinfection by using confocal laser scanning microscopy. *J Endod.* **2011**, *37*, 1380-5.	7.35	108
95	-	Verma, P.; Nosrat, A.; Kim, J.R.; Price, J.B.; Wang, P.; Bair, E.; Xu, H.H.; Fouad, A.F. Effect of residual bacteria on the outcome of pulp regeneration in vivo. *J Dent Res.* **2017**, *96*, 100-106.	7.32	51
96	62	Zehnder, M.; Schmidlin, P.; Sener, B.; Waltimo, T. Chelation in root canal therapy reconsidered. *J Endod.* **2005**, *31*, 817-20.	7.3	141
97	35	Distel, J.W.; Hatton, J.F.; Gillespie, M.J. Biofilm formation in medicated root canals. *J Endod.* **2002**, *28*, 689-93.	7.27	173
98	50	Thibodeau, B.; Trope, M. Pulp revascularization of a necrotic infected immature permanent tooth: case report and review of the literature. *Pediatr Dent.* **2007**, *29*, 47-50.	7.27	151
99	-	De-Deus, G.; Belladonna, F.G.; de Siqueira Zuolo, A., Perez, R.; Carvalho, M.S.; Souza, E.M.; Lopes, R.T.; Silva, E.J.N.L. Micro-CT comparison of XP-endo Finisher and passive ultrasonic irrigation as final irrigation protocols on the removal of accumulated hard-tissue debris from oval shaped-canals. *Clin Oral*	7.24	14

		Investig. **2019**, *23*, 3087-93.		
100	-	Althumairy, R.I.; Teixeira, F.B.; Diogenes, A. Effect of dentin conditioning with intracanal medicaments on survival of stem cells of apical papilla. *J Endod*. **2014**, *40*, 521-5.	7.23	82
-	41	Thibodeau, B.; Teixeira, F.; Yamauchi, M.; Caplan, D.J.; Trope, M. Pulp revascularization of immature dog teeth with apical periodontitis. *J Endod*. **2007**, *33*, 680-9.	7.21	163
-	44	Wilson, M. Lethal photosensitisation of oral bacteria and its potential application in the photodynamic therapy of oral infections. *Photochem Photobiol Sci*. **2004**, *3*, 412-8.	6.72	158
-	46	Chueh, L.H.; Huang, G.T. Immature teeth with periradicular periodontitis or abscess undergoing apexogenesis: a paradigm shift. *J Endod*. **2006**, *32*, 1205-13.	6.73	156
-	47	Windley, W. 3rd.; Teixeira, F.; Levin, L.; Sigurdsson, A.; Trope, M. Disinfection of immature teeth with a triple antibiotic paste. *J Endod*. **2005**, *31*, 439-43.	7.19	155
-	56	Soukos, N.S.; Chen, P.S.; Morris, J.T.; Ruggiero, K.; Abernethy, A.D.; Som, S.; Foschi, F.; Doucette, S.; Bammann, L.L.: Fontana, C.R.; Doukas, A.G.; Stashenko, P.P. Photodynamic therapy for endodontic disinfection. *J Endod*. **2006**, *32*, 979-84.	6.89	148
-	57	Peciuliene, V.; Balciuniene, I.; Eriksen, H.M.; Haapasalo, M. Isolation of Enterococcus faecalis in previously root-filled canals in a Lithuanian population. *J Endod*. **2000**, *26*, 593-5.	6.46	147
-	59	Siqueira, J.F. Jr.; Rôças, I.N.; Favieri, A.; Machado, A.G.; Gahyva, S.M.; Oliveira, J.C.; Abad, E.C. Incidence of postoperative pain after intracanal procedures based on anantimicrobial strategy. *J Endod*. **2002**, *28*, 457-60.	7.12	145
-	63	Athanassiadis, B.; Abbott, P.V.; Walsh, L.J. The use of calcium hydroxide, antibiotics and biocides as antimicrobial medicaments in Endodontics. *Aust Dent J*. **2007**, *52*(1 Suppl): S64-82.	6.73	141
-	68	Lee, S.J.; Wu M.K.; Wesselink, P.R. The effectiveness of syringe irrigation and ultrasonics to remove debris from simulated irregularities within prepared root canal walls. *Int Endod J*. **2004**, *37*, 672-8.	6.88	133
-	69	Naenni, N.; Thoma, K.; Zehnder, M. Soft tissue dissolution capacity of currently used and potential endodontic irrigants. *J Endod*. **2004**, *30*, 785-7.	6.76	133
-	71	Peters, L.B.; van Winkelhoff, A.J.; Buijs, J.F.; Wesselink, P.R. Effects of instrumentation, irrigation and dressing with calcium hydroxide on infection in pulpless teeth with periapical bone lesions. *Int Endod J*. **2002**, *35*,13-21.	6.78	132
-	72	Dunavant, T.R.; Regan, J.D.; Glickman, G.N.; Solomon, E.S.; Honeyman, A.L. Comparative evaluation of endodontic irrigants against Enterococcus faecalis biofilms. *J Endod*. **2006**, *32*, 527-31.	6.31	132
-	78	Schoop, U.; Kluger, W.; Moritz, A.; Nedjelik, N.; Georgopoulos, A.; Sperr, W. Bactericidal effect of different laser systems in the deep layers of dentin. *Lasers Surg Med*. **2004**, *35*, 111-6.	6.77	128
-	84	Rosenthal, S.; Spångberg, L.; Safavi, K. Chlorhexidine substantivity in root canal dentin. *Oral Surg Oral Med Oral Pathol Oral Radiol Endod*. **2004**, *98*, 488-92.	6.24	123
-	87	Grigoratos, D.; Knowles, J.; Ng, Y.L.; Gulabivala, K. Effect of exposing dentine to sodium hypochlorite and calcium hydroxide on its flexural strength and elastic modulus. *Int Endod J*. **2001**, *34*, 113-9.	6.86	122
-	88	Basrani, B.R.; Manek, S.; Sodhi, R.N.; Fillery, E.; Manzur, A. Interaction between sodium hypochlorite and chlorhexidine gluconate. *J Endod*. **2007**, *33*, 966-9.	6.78	121
-	89	Peters, L.B.; Wesselink, P.R. Periapical healing of endodontically treated teeth in one and two visits obturated in the presence or absence of detectable microorganisms. *Int Endod J*. **2002**, *35*, 660-7.	6.56	121
-	90	Waltimo, T.; Trope, M.; Haapasalo, M.; Ørstavik, D. Clinical efficacy of treatment procedures in endodontic infection control and one year follow-up of periapical healing. *J Endod*. **2005**, *31*, 863-6.	5.63	121

-	93	Chueh, L.H.; Ho, Y.C.; Kuo, T.C.; Lai, W.H.; Chen, Y.H.; Chiang, C.P. Regenerative endodontic treatment for necrotic immature permanent teeth. *J Endod*. **2009**, *35*, 160-4.	6.44	118
-	95	Estrela, C.; Sydney, G.B.; Bammann, L.L.; Felippe Júnior, O. Mechanism of action of calcium and hydroxyl ions of calcium hydroxide on tissue and bacteria. *Braz Dent J*. **1995**, *6*, 85-90	7.08	115
-	96	Teixeira, C.S.; Felippe, M.C.; Felippe, W.T. The effect of application time of EDTA and NaOCl on intracanal smear layer removal: an SEM analysis. *Int Endod J*. **2005**, *38*, 285-90.	6.2	115
-	97	Portenier, I.; Haapasalo, H.; Rye, A.; Waltimo, T.; Ørstavik, D.; Haapasalo, M. Inactivation of root canal medicaments by dentine, hydroxylapatite and bovine serum albumin. *Int Endod J*. **2001**, *34*, 184-8.	5.85	115
-	99	Sunde, P.T.; Olsen, I.; Debelian, G.J.; Tronstad, L. Microbiota of periapical lesions refractory to endodontic therapy. *J Endod*. **2002**, *28*, 304-10.	5.04	112
-	100	Spratt, D.A.; Pratten, J.; Wilson, M.; Gulabivala, K. An in vitro evaluation of the antimicrobial efficacy of irrigants on biofilms of root canal isolates. *Int Endod J*. **2001**, *34*, 300-7.	5.37	111
76 articles were common to the top 100 RCRs and top 100 cited.				
