# Supplementary material for: Root Canal Disinfection Articles with the Highest Relative Citation Ratios. A Bibliometric Analysis from 1990 to 2019
Source: Antibiotics (Basel). 2021 Nov 18;10(11):1412. doi: 10.3390/antibiotics10111412 (PMC8614753; doi:10.3390/antibiotics10111412)
Supplement: Supplementary file 1 [file antibiotics-10-01412-s001.zip › Supplementary materials Table S2.pdf]

Table S2. Keywords with at least three co-ocurrences among the top 100 RCR articles in root canal disinfection

**Keywords**	**Co-occurrences**	**Total link strengh**
root canal irrigants	91	887
sodium hypochlorite	52	559
dental pulp cavity	44	473
dentin	36	347
root canal preparation	36	395
root canal therapy	27	298
chlorhexidine	26	271
calcium hydroxide	24	258
periapical periodontitis	22	304
anti-infective agents, local	21	213
drug combinations	21	287
smear layer	21	190
edetic acid	20	214
tooth apex	18	298
dental pulp necrosis	16	268
enterococcus faecalis	16	144
female	16	261
male	16	267
anti-bacterial agents	13	200
microscopy, electron, scanning	13	120
root canal filling materials	13	234
dental pulp	12	168
follow-up studies	12	221
time factors	12	130
bacteria	11	119
biofilms	11	113
child	11	183
metronidazole	11	181
therapeutic irrigation	11	72
colony count, microbial	10	108
treatment outcome	10	167
adult	9	131
analysis of variance	9	87
animals	9	78
ciprofloxacin	9	151
disinfectants	9	109
microbial sensitivity tests	9	78
neovascularization, physiologic	9	143
adolescent	8	119
aluminum compounds	8	145
anti-infective agents	8	109
bacteria, anaerobic	8	72
calcium compounds	8	145

chelating agents	8	96
citric acid	8	85
minocycline	8	139
oxides	8	145
silicates	8	145
tooth root	8	92
apexification	7	115
dental disinfectants	7	70
molar	7	88
regeneration	7	77
statistics, nonparametric	7	62
ultrasonics	7	44
bicuspid	6	83
disinfection	6	79
middle aged	6	84
periapical tissue	6	101
tissue scaffolds	6	76
bacterial infections	5	44
chi-square distribution	5	56
dentin permeability	5	41
equipment design	5	76
incisor	5	78
microscopy, confocal	5	52
odontogenesis	5	114
sodium chloride	5	56
statistics as topic	5	73
cattle	4	41
cell survival	4	56
cells, cultured	4	56
dental alloys	4	78
detergents	4	52
dose-response relationship, drug	4	51
doxycycline	4	51
gels	4	40
nickel	4	78
periapical diseases	4	56
pharmaceutical vehicles	4	29
polysorbates	4	51
regenerative endodontics	4	46
retreatment	4	46
stem cells	4	55
tissue engineering	4	42
titanium	4	78
tooth, nonvital	4	43
water	4	40
zinc oxide-eugenol cement	4	68

aged	3	56
biocompatible materials	3	42
calcium	3	39
dental leakage	3	26
dental papilla	3	41
gutta-percha	3	64
hydrogen peroxide	3	24
image processing, computer-assisted	3	39
intercellular signaling peptides and proteins	3	27
linear models	3	41
needles	3	18
platelet-rich plasma	3	46
potassium iodide	3	20
radiography	3	55
solutions	3	28
syringes	3	20
x-ray microtomography	3	19
